# Embryonic Demise Caused by Targeted Disruption of a Cysteine Protease *Dub-2*

**DOI:** 10.1371/journal.pone.0044223

**Published:** 2012-09-12

**Authors:** Kwang-Hyun Baek, Heyjin Lee, Sunmee Yang, Soo-Bin Lim, Wonwoo Lee, Jeoung Eun Lee, Jung-Jin Lim, Kisun Jun, Dong-Ryul Lee, Young Chung

**Affiliations:** Department of Biomedical Science, CHA Stem Cell Institute, CHA University, CHA General Hospital, Gyeonggi-Do, Republic of Korea; Mayo Clinic, United States of America

## Abstract

**Background:**

A plethora of biological metabolisms are regulated by the mechanisms of ubiquitination, wherein this process is balanced with the action of deubiquitination system. *Dub-2* is an IL-2-inducible, immediate-early gene that encodes a deubiquitinating enzyme with growth regulatory activity. DUB-2 presumably removes ubiquitin from ubiquitin-conjugated target proteins regulating ubiquitin-mediated proteolysis, but its specific target proteins are unknown yet.

**Methodology/Principal Findings:**

To elucidate the functional role of *Dub-2*, we generated genetically modified mice by introducing neo cassette into the second exon of *Dub-2* and then homologous recombination was done to completely abrogate the activity of DUB-2 proteins. We generated *Dub-2*+/− heterozygous mice showing a normal phenotype and are fertile, whereas new born mouse of *Dub-2*−/− homozygous alleles could not survive. In addition, *Dub-2*−/− embryo could not be seen between E6.5 and E12.5 stages. Furthermore, the number of embryos showing normal embryonic development for further stages is decreased in heterozygotes. Even embryonic stem cells from inner cell mass of *Dub-2*−/− embryos could not be established.

**Conclusions:**

Our study suggests that the targeted disruption of *Dub-2* may cause embryonic lethality during early gestation, possibly due to the failure of cell proliferation during hatching process.

## Introduction

Ubiquitination and deubiquitination are important for post-translational modification and responsible for many intracellular processes, including cell cycle progression, transcriptional activation and signal transduction [1]–[3]. Most of these cellular functions are relayed on the subsequent degradation of proteins through 26S proteasomal pathway [4], [5]. Ubiquitination is a well-established event involving enzyme cascade such as ubiquitin-activating enzymes (E1), ubiquitin-conjugating enzymes (E2), and ubiquitin ligase (E3) enzymes that mediate attachment of ubiquitin molecules to the lysine residues in proteins targeted to the 26S proteasome for degradation [1], [6], [7]. Deubiquitinating enzymes (DUB) are one of cysteine protease families and act to counterbalance ubiquitination event by cleaving ubiquitin from ubiquitin-conjugated protein substrates [1], [2], [8]. There are at least five major families of DUB enzymes, UBP or USP (ubiquitin-specific processing proteases) [9], UCH (ubiquitin carboxy terminal hydrolases) [9], JAMM (Jad1/Pad/MPN-domain-containing metallo enzymes) [10], OTU (Otu-domain ubiquitin-aldehyde-binding proteins) [11] and Ataxin-3/Josephin [12], [13]. The process of ubiquitination and deubiquitination are disturbed by several factors leading to tumorigenesis [14]–[16].

Murine DUB enzymes have been identified and belong to members of UBP or USP subfamily and have cellular growth regulatory activity [2], [17]–[21]. Murine DUB subfamily may consist of four to six highly homologous enzymes and each shows extensive homology (approximately 90%) except the hypervariable region that may allow substrate specificity [2], [8]. *Dub-1* is an immediate early gene, which is induced by interleukin-3 (IL-3), interleukin-5 (IL-5), or granulocyte-macrophage colony stimulating factor (GM-CSF) in Ba/F3 (pro-B cell line) [2], [19], [20]. *Dub-2* is also an immediate early gene, which is induced by IL-2 in CTLL-2 (murine cytotoxic T lymphocyte) [2], [21]. It is interesting to find that mRNAs for both *Dub-1* and *Dub-2* are induced rapidly in response to IL-3 and IL-2 stimulation, respectively, but rapidly decline with depletion [19]–[21]. *Dub-3*, which is known as *Usp17*, is responsible for the regulation of cell growth and survival, and the constitutive expression of *Dub-3* can block cell proliferation [22]–[25]. A broad range of proteins are involved in the ubiquitin-proteasome system (UPS) so that any abnormality in related proteins could suffer pathogenesis for a number of diseases. For example, cystic fibrosis, Angelman’s syndrome and Liddle syndrome can arise because related proteins are too stable or too accelerating for degradation [26]. In addition, aberrant regulation of ubiquitination system is involved in immune diseases, neurodegenerative diseases, and muscle wasting [27], [28].

In our study, we generated *Dub-2* knock-out mice by targeting gene. As a functional consequence, homozygous mice seem to be lethal during pre-implantation stage. Like survivin, an anti-apoptotic factor expressed in pre-implantation embryo [29], *Dub-2* is also expressed during early developmental stage, and disruption causes embryonic lethality. It has been demonstrated that proteasomes play an essential role in thymocyte apoptosis and *Dub-2* expressed specifically in T-lymphocytes in response to IL-2 [20] may be involved in anti-apoptotic progression during the early embryonic development [30]–[32]. A previous report demonstrated that constitutive expression of *Dub-2* prolongs the cytokine-induced Jak/STAT signaling events and delays the apoptotic process that follows cytokine withdrawal [18]. Here, we provide a significant function of *Dub-2* during early embryonic development, which may be required for hatching and development of inner cell mass (ICM).

## Results and Discussion

### Generation of *Dub-2* Mutant Mice

**Figure 1:**
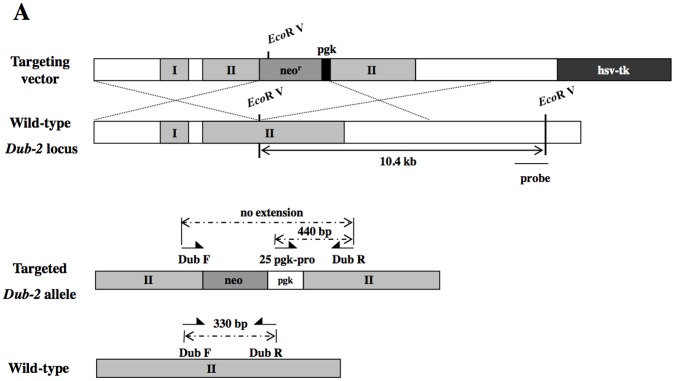
Targeted disruption of the *Dub-2* gene. **A)** Schematic representation of the targeting vector and the targeted allele of *Dub-2* gene. *Dub-2* is composed of two exons and exon II is disrupted in a targeting vector. Neo^r^ and hsv-tk are used as positive and negative selection markers, respectively. The probe used for Southern hybridization is shown as a black bar. Two pairs of arrows below wild-type and targeted alleles indicate the positions of the primers used for genotyping. **B)** Southern blot analysis of genomic DNA from targeted *Dub-2* ES cells with the probe to distinguish targeted (12.2 kb) allele from wild-type (10.4 kb) allele. **C)** Genotyping by genomic PCR. The 440 bp band is amplified by using three kinds of primer (Dub F, Dub R, and 25 pgk pro). In addition, PCR analysis of genomic DNA extracted from wild-type and *Dub-2*+/− ES cell clones was performed. The amplified fragments derived from wild-type and mutant alleles are indicated.

To investigate the physiological roles of *Dub-2* in mouse, we generated *Dub-2* knock-out mice. Mouse *Dub-2* is encoded by 2 exons that expand approximately 15 kb long genomic DNA. The targeted mutagenesis strategy for *Dub-2* included the insertion of a neo-expression cassette, which abolishes the translation of the catalytic domain of the enzyme [2], [8] (**Figure 1A**). Southern blot and genomic PCR screenings identified 4 clones as having a targeted *Dub-2* allele (**Figures 1B and 1C**). ES cells from clone 13 were injected into C57BL/J6 blastocysts which generated several chimeric mice. 3 chimeric males transmitted the targeted *Dub-2* allele to the offsprings of C57BL/6J (outbred) and 129SV/J (inbred) females were obtained. Germline transmission of this allele was further confirmed by the identification of both heterozygous males and females which were used as a breeding pair. When *Dub-2* heterozygotes were intercrossed, no *Dub-2*−/− animals were observed among 215 mice genotyped at the age of 4 weeks (**Figure 1C** **and** **Table 1**), indicating that the absence of *Dub-2* causes embryonic lethality.

**Table 1:** Genotyping analysis for different stages of embryos.

Stage	Genotype				
	Dub-2+/+	Dub-2+/−	Dub-2−/−	Total	ND
4 wks	54	161	0	215	
11–12dpc	5	4	0	9	
10.5dpc	3	5	0	8	
7.5dpc	23	60	0	88	5
6.5dpc	10	45	0	65	10
3.5dpc	75		13	95	7
2.5dpc	7		7	16	2
1.5dpc	42		3	50	5
0.5dpc	35		10	47	2

### Embryonic Lethality of *Dub-2*−/− Mice

The heterozygous mutant mice, which were fertile, showed phenotypically normal in their development and global anatomy. Intercrosses between heterozygotes produced no viable offspring homozygous for the mutant *Dub-2* allele (*Dub-2−/−*) (**Table 1**). This indicates that *Dub-2* expression is required for the survival and development of embryos. Timed pregnancies of *Dub-2*+/− heterozygote matings were analyzed between E6.5 and E12.5 of gestation stage in order to determine the developmental stage of embryos where the embryos become lethal. *Dub-2*−/− embryos were not shown in these stages (**Table 1**). Therefore, we analyzed embryos at the stage of E0.5, E1.5, E2.5 and E3.5 for the presence of homozygotes. Interestingly, homozygous embryos were detected until the developmental stage of E3.5 (**Table 1**). 13 of 95 blastocysts isolated at E3.5 were homozygous and 75 were either wild-type or heterozygous for the targeted *Dub-2* allele. The blastocysts displayed normal morphology with zona pellucida, inner cell mass, and trophectoderm. *Dub-2* deficient embryos die before implantation at the stage when blastocysts hatch from their zona pellucida. None of 65 embryos with this morphology turned out to be of the homozygous (*Dub-2*−/−), indicating that the embryonic lethality occurs at the early stage of preimplantation. Like anti-apoptotic factors expressed in pre-implantation embryo [29], *Dub-2* is also expressed during early embryogenesis and disruption causes embryonic lethality.

### Analysis of Heterozygous Mice

**Figure 2:**
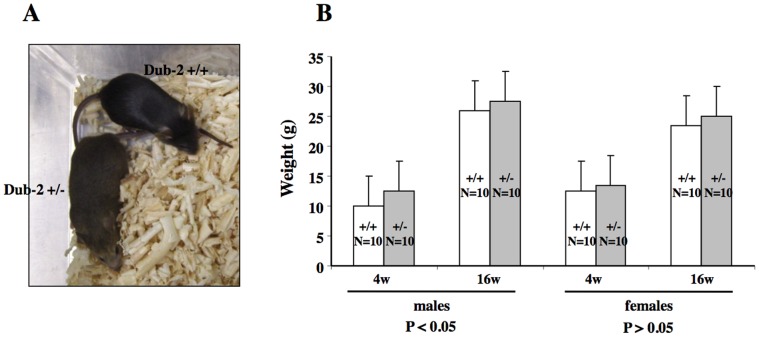
The phenotypes of *Dub-2*-deficient mice. **A)** Representative 8 week old mice of the indicated genotype demonstrating a size difference in heterozygous males. **B)** Mice of the indicated genotype were weighed at the indicate ages in weeks. **C)** The weight of ovary, testis, thymus, and spleen was measured. While there is no difference for the ovary, statistically significant difference was observed for the testis. The weight of the thymus in *Dub-2+/−* is heavier than that of *Dub-2+/+* mice.

We observed differences in size between *Dub-2*+/+ and *Dub-2*+/− littermates (**Figure 2A**). Although heterozygous mice seemed to be normal at birth and had no apparent developmental defect by histological analyses, the mean body weight of *Dub-2*+/− mice was significantly higher than that of wild-type mice (**Figure 2B**). The weight difference developed within the first 4 weeks after birth as heterozygous mice weighed approximately 120% of wild-type mice. The weight of most organs in *Dub-2*+/− mice is similar to those of wild-type mice but there was a significant weight difference in the testis (**Figure 2C**).

**Figure 3:**
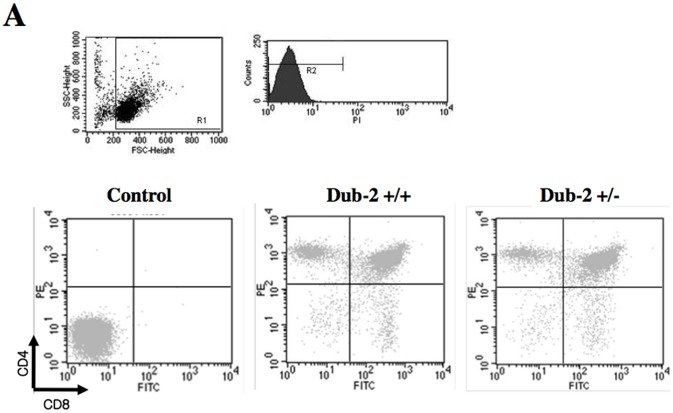
Lymphoid cell population in the thymus and spleen of 5 weeks old animals was analyzed by FACS. **A)** Thymocytes were harvested from *Dub-2*+/+ and *Dub-2*+/− mice with the indicated genotypes, fixed with ethanol, and stained with FITC-conjugated anti-CD8, and PE-conjugated anti-CD4. **B)** The graph shows the ratio of lymphoid cells with CD4+ or CD8+. The ratio of thymocytes from *Dub-2+/+* was calibrated as 100%. **C)** Splenocytes were harvested from *Dub-2*+/+ and *Dub-2*+/− mice with the indicated genotypes, fixed with ethanol, and stained with FITC-conjugated anti-B220, and APC-conjugated anti-CD3. D) The graph shows the ratio of lymphoid cells with CD3+ or B220+.

Since *Dub-2* is expressed only in T-lymphocytes [2], [13], we analyzed thymocytes and splenocytes of 5-week-old littermates for T-lymphocyte and B-lymphocyte development by flow cytometry. Thymocytes and splenocytes were analyzed using antibodies against CD4, CD8, CD3 and B220. The total number of lymphocytes in thymus and spleen was significantly different between *Dub-2*+/+ and *Dub-2+/−* (**Figure 3** **)**. In thymus of *Dub-2+/−* animals, the number of CD8+ and CD4+ T cells was increased by 40% and 45%, respectively (**Figures 3A and 3B**). In spleen of *Dub-2+/−* animals, the number of B220+ B cell was increased by 4-fold but CD3+ T cell was decreased by 3-fold (**Figures 3C and 3D**). Since it has been known that *Dub-2* is expressed only in T-lymphocytes [21], important directions for further studies will include analysis of T-lymphocyte and B-lymphocyte development in the absence of *Dub-2* gene.

### *Dub-2* Null Embryos Fail to Hatch *in vitro*

**Figure 4:**
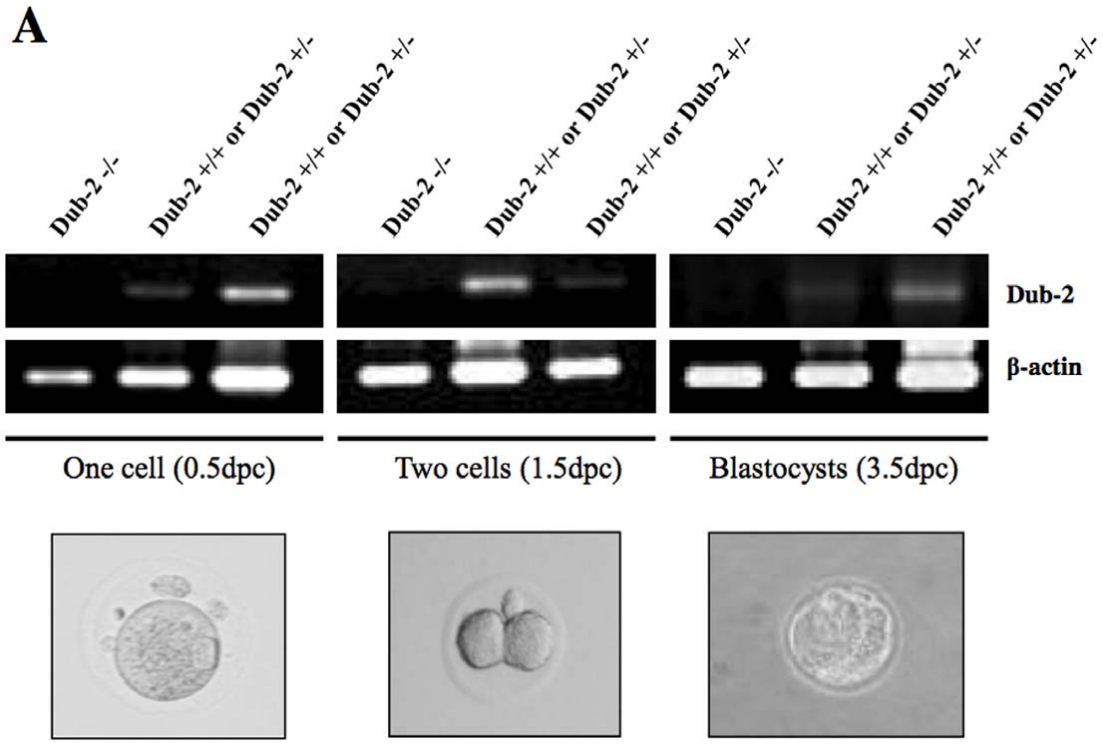
*Dub-2*−/− embryos fail to hatch *in vitro*. **A)** Genotyping by genomic PCR. 330 bp, amplified by using Dub F and Dub R primers, represents either *Dub-2+/+* or *Dub-2+/−*, whereas no band represents *Dub-2−/−*. *Dub-2*−/− embryos were found between 0.5dpc and 3.5dpc. **B)** Out-growth inner cell mass was harvested and then genotype was confirmed by 2-primers PCR (Dub F and Dub R) as described in Materials and Methods. Developmental defects in *Dub-2*−/− embryos at the 5.5dpc stage were shown *in vitro*. ICM grew normally and was surrounded by trophoblast giant cells in *Dub-2+/+* and *Dub-2*+/− embryos. In contrast, *Dub-2*−/−5.5dpc embryos did not hatch to the culture dish. **C)** The number of hatching status of 3.5dpc embryos after 96 hrs of culture *in vitro*. *Dub-2* deficiency leads to failure to hatch *in vitro*. **D)** Analysis of TUNEL assay was performed for cell death. Arrows pointing spots indicate cells undergoing apoptosis. **E)** E3.5 blastocysts were isolated, and after 24 hrs in culture, their ZP was mechanically removed. The ZP-free embryos were cultured on feeder layer in ES cell medium. **F)** *Dub-2*+/+ and *Dub-2*+/− mES cell lines.

Out of 215 pups born, the ratio of wild-type to heterozygous to null genotypes was about 1∶3:0 (**Table 1**). The *Dub-2+/−* mice used for the intercross had been backcrossed to the C57BL/J6 background for the three or more generations, arguing against the possibility that the embryonic lethal phenotype was due to other nonspecific mutations. Interestingly, *Dub-2−/−* embryos can be identified at the blastocyst stage, as was shown by the nested PCR genotyping of isolated E0.5 to E3.5 blastocysts (**Figure 4A**). The expression level of *Dub-2* was not changed between E0.5 and E3.5 (data not shown). Genomic PCR analysis revealed that *Dub-2−/−* embryos were found between E0.5 and E3.5. However, no embryo bearing the null genotype was recovered after E5.5 stage (**Figure 4B** **,** **Table 1**). Heterozygous embryos appeared as fully expanded blastocysts at the time of isolation, and following hatching, expansion of both inner cell mass (ICM) and trophectoderm (TE) cell types was evident after 48 hrs of culture *in vitro*, giving rise to adherent sheets of trophoblastic giant cells and outgrowth of the ICM (**Figure 4C**). The remaining embryos failed to hatch are likely to be homozygotes (**Figure 4C** **and** **Table 2**). As shown in **Figure 4**, *Dub-2* deficiency leads to failure to hatch *in vitro*. Hatching process *in vitro* requires active participation of the trophectoderm. TUNEL assays further revealed apoptosis of the trophectoderm in the *Dub-2* null embryos, whereas heterozygous and wild-type outgrowths developed negative level of apoptosis (**Figures 4D and 4E**). These data indicate that the loss of *Dub-2* function results in early embryonic lethality during preimplantation, and that *Dub-2* may play an essential role in hatching process regulated by the trophectoderm.

**Table 2:** The number of hatching status of E3.5 embryos after 96 hrs of culture *in vitro*.

Genotype	Number of embryos	Number of hatched
Dub-2+/+	10	10
Dub-2+/−	45	45
Dub-2−/−	13	0
ND	12	0

Hatching process can take place with fewer cells at a time point that may reflect the onset of blastocyst expansion. Importance of hatching enzymes in zona shedding of mouse blastocysts rather than the mechanical movement of embryos becomes clear. Hatching enzymes play a role on the surface of the plasma membrane in blastomeres and are secreted to the peri-vitelline space where they function during the hatching process. Therefore, *in vivo* hatching may occur simultaneously from both inside and outside the blastocyst [33]. Our current study suggests that aberrant rupture of embryos may occur due to the failure of hatching as a result of *Dub-2* knock-out during the blastocysts stage. Thus, deficiency in processing and secretion of hatching enzymes (e.g. peptidyl argininal, progesterone, and trypsin like proteinase) [33]–[35], which may be regulated by DUB-2 enzyme, can obstruct *Dub-2*−/− embryos from hatching and expanding of blastocysts, due to the fact that the percentage of blastocyte formation in a given period of time (84 hrs) was not affected by *Dub-2*+/− heterozygous gametes.

### *Dub-2* is Required for Embryonic Cell Proliferation and Protecting the Inner Cell Mass from Apoptosis

We then tested whether *Dub-2*−/− embryos are viable in their blastocyst stage. To test this, the zona pellucida (ZP) of the E3.5 embryos from the *Dub-2*+/− intercross was mechanically removed. These ZP-free embryos were then cultured according to ES cell culture method and examined for their growth, which was followed by PCR genotyping. As expected, wild-type, and heterozygous *Dub-2*+/− embryos expanded healthy in culture, giving rise to both the ICM and TE cell types (**Table 3**). And then a line of ES cell was successfully established. (**Figure 4F**) However, the homozygous *Dub-2*−/− embryos were unable to expand in long term culture even after 9.5 days of culture and appeared degenerative. Thus, the lack of cell expansion of the homozygous *Dub-2* null embryos is likely due to combined effect of a general proliferation defect and an increase in apoptosis of the ICM.

**Table 3:** The number of hatching status of E3.5 embryos after 114 hrs of ZP-free culture *in vitro*.

Genotype	Number of zona free embryo	Number of initial outgrowth		Number of ES cells
		For genotyping	For ES cell establishment	
Dub-2+/+	132	9	55	9
Dub-2+/−		25		21
Dub-2−/−		10		0
ND	22	15	18	15
Total	154	59	73	55

It is possible that apoptosis in pre-implantation embryos occurs due to chromosomal and nuclear abnormalities, including multiploidy and mosaicism [36], [37]. Inappropriate developmental potential can also lead to apoptosis due to the aberrant presence of ICM with the trophectoderm and/or the imbalance of growth or survival factors [38]. It could also be the involvement of the different pro- and anti-apoptotic molecules associated with DUBs that are disturbed during early embryonic development. Several evidences show that germ cells and germ cell tumors signal for the process of cell death [39]. Recent studies on *HAUSP/Usp7* knock-out mice also showed similar embryonic lethality during early embryonic development and died between embryonic days E6.5 and E7.5 [40]. Recently, it was reported that germ cell apoptosis is regulated by a DUB enzyme CYLD [41], suggesting that the ubiquitin-proteasome pathway plays a critical role in early development, as shown with the DUB-2 enzyme in this study.

### Effect of *Dub-2* on Mouse Sperm Survival and *in vitro* Fertilization

**Figure 5:**
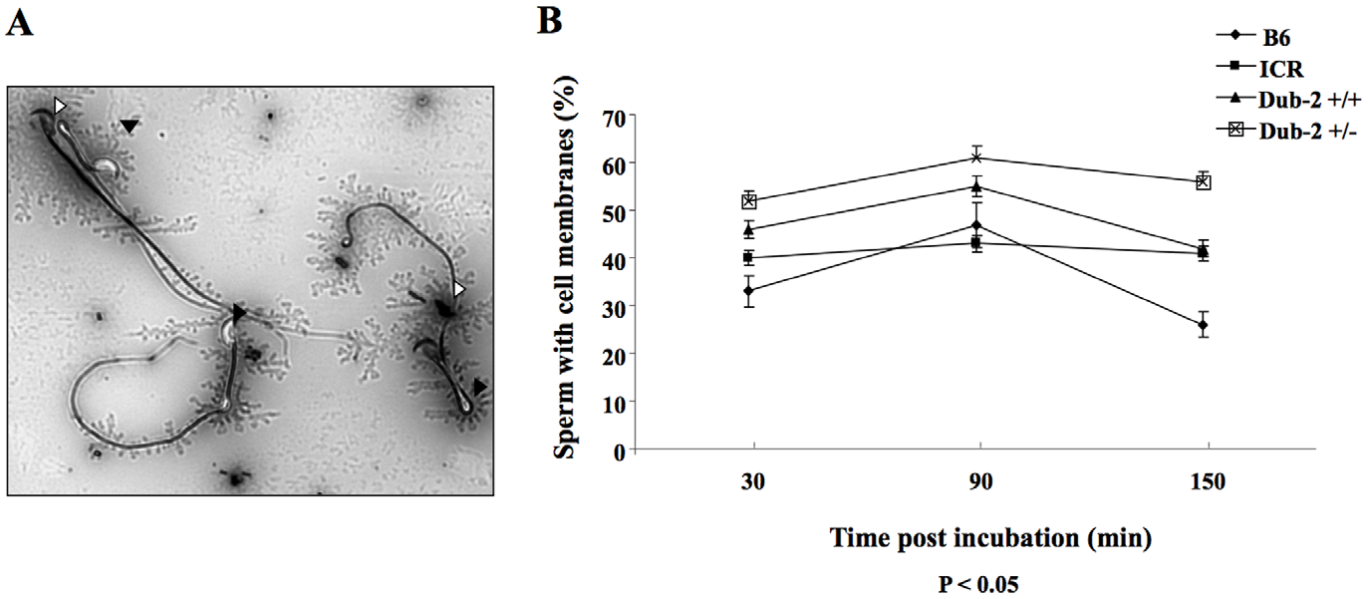
Analysis of sperm survival. **A)** Vital staining of sperms. White arrows indicate dead sperms and black arrows indicate survival sperms. **B)** Percentages of mouse sperm with intact cell membranes for B6 mouse, ICR, 129SV, *Dub-2*+/+, and *Dub-2*+/−. *Dub-2*+/− group appeared to have lower sperm survival as identified by the vital staining after time post incubation.

**Figure 6:**
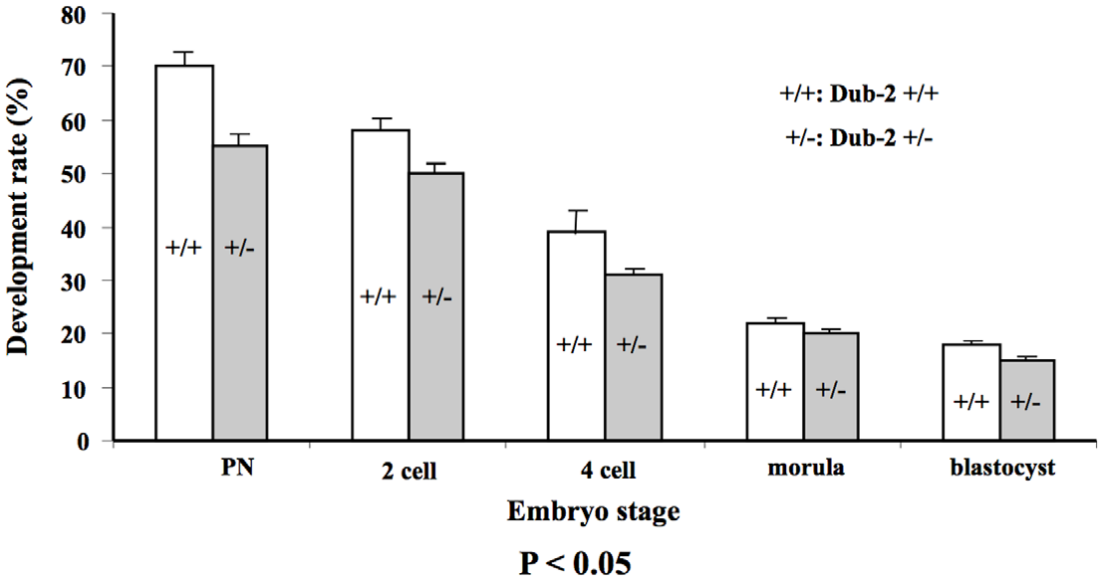
Effect of *Dub-2*+/− heterozygous gamete (oocyte and sperm) on *in vitro* fertilization and embryo development. *In vitro* fertilized zygotes were cultured for 96 hrs (post hCG) in the basic culture media and scored at subsequent preimplantation stages (post hCG 48 hrs, 65 hrs, 72 hrs, and 96 hrs). Experiments were performed three time and the data are expressed as percentage ± SEM.

While the mean weight of ovary in *Dub-2+/−* mice is similar to that of ovary in wild-type mice (**Figure 2C**), the mean weight of testis in *Dub-2+/−* mice is significantly lower compared with that of testis in wild-type mice **(** **Figure 2C** **).** In addition, vital staining showed significant differences in the percentage of sperm with intact membranes at 30, 90, and 150 min post-incubation (**Figures 5A and 5B**). The higher percentage of cell membrane was shown in *Dub-2+/−* sperms compared with *Dub-2+/+* (**Figure 5B**). However, motility and progressive movement in the *Dub-2+/−* sperm seemed to be lower than that in the *Dub-2+/+* (positive control) or other control mice including C57BL/J6 and ICR (**Table 4**). In order to investigate whether sperms from *Dub-2+/−* mice have comparable fertilization capability to ones from wild-type mice, *in vitro* fertilization was performed. Fertilization *in vitro* was successful, as on average 60% of the oocytes cleaved to the 2-cell stage 21 hrs after following insemination. The percentage of embryos cleaving to 2- to 4-cell, morula, and blastocyst in the basic culture media are shown in **Figure 6**. The rate of pronuclear formation and early cleavage stage embryos was lower (PN: 55.5% *vs* 69.9%, 2-cell: 49.5% *vs* 56.8%, and 4-cell: 29.9% *vs* 37.8%). However, the percentage of blastocyte formation in a given period of time (84 hrs) was not affected by *Dub-2+/−* heterozygous gametes. During *in vitro* culture, no parthenogenetical activation was observed in either group.

**Table 4:** Motility and progressive movement of mouse spermatozoa.

Sperm group	Motile sperm (%)			Progressive movement score		
	30 min	90 min	150 min	30 min	90 min	150 min
B6	39.6	55	28.6	23.76	33	17.16
ICR	59.4	50.6	48.4	35.64	30.36	29.04
Dub-2+/+	52.8	62.0	46.5	31.68	37.2	27.9
Dub-2+/−	28.6	39	19.1	17.16	23.4	11.46

Our study suggests that the targeted disruption of *Dub-2* may cause embryonic lethality during early gestation, possibly due to the failure of cell proliferation during hatching process. We found that cell proliferation in the ICM is substantially reduced in homozygous *Dub-2* null embryos, indicating that DUB-2 and its substrate proteins are essential for cell proliferation. Therefore, further experiments will be required in determining the role of DUB-2 in hatching process and proliferation of embryonic cells during pre-implantation.

## Materials and Methods

### Construction of a Targeting Vector and Gene Targeting of *Dub-2* in ES Cells

The *Dub-2* targeting vector was generated from genomic DNA fragments derived from a mouse 129w genomic clone, Clone 4. The Clone 4 was identified by screening a mouse 129w strain genomic library by PCR (Genome Systems, St. Louis, MO, USA) (**Figure 1A**). Exon 2 was interrupted by a replacement with *Neo^R^* (neomycin) and *hsv-tk* (thymidine kinase) used for positive and negative selection markers, and was transfected into mouse ES cells (129/SvEv) from ATCC. The targeted ES cells were used to generate mice with a null mutant of *Dub-2*. Screening for recombinant ES cell clones was performed by G418 selection (350 µg/ml) for 7 days. Surviving ES cell colonies were picked and expanded for Southern blot analysis. The probe used for Southern hybridization is shown as a black bar (**Figure 1A**). Two pairs of arrows below wild-type and targeted alleles indicate the positions of the primers used for genotyping (**Figure 1A**).

### Generation of *Dub-2* Knock-out Mice

ES cells were electroporated with the linearized targeting construct and were cultured. For genotyping ES cell clones, DNA was digested with *EcoR* V, fractionated on agarose gels, and subjected to Southern blot analysis by using the ^32^P-labeled probe. The sizes of expected *Dub-2* fragments are shown (**Figure 1B**). To obtain chimeric mice, the ES cell clones that contain a PGK-disrupted *Dub-2* allele were injected into mouse blastocysts. Among the pups produced, four male chimers were identified and bred with C57BL/J6 females to generate *Dub-2*+/− C57/129 hybrid mice or with 129w females to produce 129-inbred mice. The *Dub-2*+/− hybrid F1 progeny was either interbred or bred with corresponding 129w mice. Genotyping of mice was performed by standard Southern blot analysis of tail DNA as described above. For PCR-based genotyping, a forward primer for the disrupted allele which was designed from the complementary sequence of the PGK promoter (25 pgk-pro: TTC CTG ACT AGG GGA GGA GTA GAA G) or a forward wild-type allele specific primer (Dub F: TTG GAG GCT TGT GGA GGT CTC AGA T) from the 2 kb *BamH* I fragment that was deleted in the disrupted allele, and a common reverse primer (Dub R: GAC AGG TAT GGC TTC AGG TCA AGG A) from the 3′ *Xho* I fragment were used in the generation of the disruption construct (**Figure 1A**). Two separate PCRs were performed, one for wild-type allele and the other for the disrupted allele, using 35 cycles of amplification, with each cycle containing incubation at 95°C for 1 min, 60°C for 30 sec, and 72°C for 1 min, after an initial 2 min, 94°C denaturation. These primers amplify 440- and 330-bp products that are specific to knock-out allele and wild-type allele, respectively. All animal procedures were approved by Institutional Animal Care and Use Committee at CHA University as required.

### Genotyping Analysis of Embryos

Blastocysts were collected at embryonic development date 3.5 (E3.5) by flushing the uterus with M2 medium (Sigma, St. Louis, MO, USA). These blastocysts were then transferred to an ES medium in a 0.1% gelatin treated 96 well culture dish and cultured at 37°C with 5% CO_2_. After photographing the blastocyst outgrowth, colonies were lysed in a buffer containing 10 mM Tris-HCl (pH 8.5), 50 mM KCl, 2 mM MgCl_2_. 0.45% NP40, 0.45% Tween 20, and 60 µg of proteinase K per ml. The samples were boiled for 10 min to inactivate the protease. A half of the lysate was used for 25 µl PCR, starting with denaturing at 94°C for 5 min, followed by 34 cycles of 94°C for 30 sec, 60°C for 30 sec, and 72°C for 10 min. The PCR primers, 25 pgk-pro, Dub F, and Dub R, were used.

For genotyping analysis at the early stage of embryos, blastocysts were collected at embryonic development date 0.5 (E0.5), 1.5 (E1.5) and 3.5 (E3.5) by flushing the uterus and oviduct with M2 medium (Sigma, St. Louis, MO, USA). Single blastocyst was collected in 1.5 ml Eppendorf test tubes and Dynabeads DNA Direct Universal buffer (Invitrogen, Carlsbad, CA, USA) was added. A half of the lysate was used for 10 µl PCR, starting with denaturing at 94°C for 5 min, followed by 34 cycles of 94°C for 30 sec, 60°C for 30 sec, and 72°C for 10 min. The PCR primers, Dub F and Dub R, were used with denaturing at 94°C for 5 min, followed by 34 cycles of 94°C for 30 sec, 60°C for 30 sec, and 72°C for 10 min. The PCR primers for controls, β-actinF (5′ CGA GCG GTT CCG ATG CCC TGA G 3′), β-actinR (5′ GGC CGG ACT CAT CGT ACT CCT G 3′), and GAPDH F (5′ TGT TCC TAC CCC CAA TGT GT 3′), and GAPDH R (5′ TGT GAG GGA GAT GCT CAG TG 3′) were used.

### TUNEL Assay

The blastocysts were cultured for 48 hrs *in vitro*, fixed in 4% paraformaldehyde for 30 min at room temperature, and permeablized (0.1% Triton X-100 and 1% bovine serum albumin in PBS) for 10 min. After incubation with 100 µl TUNEL reaction mixture (Biotin-dUTP, Roche-Applied Science, Mannheim, Germany) at 37°C for 2 hrs, and washed three times with PBS. The embryos (5.5 dpc) were incubated with anti-streptavidin-HRP for 1 hr at room temperature. Reaction mixtures were developed with 3,3-diaminobenzidinetetrahydrochloride.

### mES Cell Line Derivation from *Dub-2* Knock-out Mice Embryos

Two-cell embryos were collected from female *Dub-2+/−* knock-out mice mated with *Dub-2+/−* knock-out males at 44∼46 hrs after human chorionic gonadotrophin (hCG) injection, and cultured in P1 (Irvine Scientific Inc. Santa Ana, CA, USA) medium supplemented with 10% Synthetic Serum Substitute (Irvine Scientific Inc. Santa Ana, CA, USA) at 37°C with 5% CO_2_ in air to blastocyst stage. To get outgrowths, all of morulaes and blastocysts were removed using 0.5% pronase (Sigma, St. Louis, MO) in P1 and then placed on mitotic inactivated MEF feeder cells in mES culture medium. We used DMEM/F12 containing 20% Knock-out SR, 0.1 mM β-mercaptoethanol, 1% nonessential amino acids, 100 units/ml penicillin, 100 µg/ml streptomycin (all products from Gibco/Invitrogen, Grand Island, NY, USA), and 1.5×10^3^ units/ml rmLIF (Chemicon, Temecula, CA, USA) for mES cell culture medium. After 3∼4 days, all outgrowths were collected for PCR analysis of genomic DNA. To establish mES cell lines, morulaes and blastocysts except partially hatched blastocysts were removed using 0.5% proteinase in P1 and then placed on mitotic inactivated MEF feeder cells in mES culture medium to form outgrowth. Growing outgrowths were transferred on new MEF feeder cells mechanically at first and then passaged using trypsin-EDTA. All of established mES cell lines were analyzed by genomic DNA PCR to confirm their status for *Dub-2+/−* gene such as wild-type, heterozygote or homozygote.

### *In vitro* Fertilization

*Dub-2* heterozygote and wild-type males with proven fertility were sacrificed by cervical dislocation and the epididymides and vas deferens were removed and placed in 1 ml of tubal fluid medium supplemented with 0.5% BSA. The epididymides were punctured with a needle and sperms were removed from the vas deferens. The suspension of sperms was placed in a humidified incubator at 37°C, 5% CO_2_ for 1 hr in order to capacitate. The oocyte cumulus cell complexes were retrieved from 6–8 weeks old *Dub-2* heterozygote and wild-type females after 14 hrs of hCG injection. The oocyte cumulus cell complexes were placed in 500 µl tubal fluid supplemented with 0.5% BSA to which 40 µl of capacitated sperm suspension was added. The fertilization dishes were incubated in a humidified environment at 37°C, 5% CO_2_ for 5 hrs, at which the oocytes were washed free of sperm through several microdrops of tubal fluid and potassium simplex-optimizsed (KSOM) medium, and were transferred to KSOM. The oocytes were cultured overnight and assessed for 2-cell cleavage the following morning. A subset of these embryos was analyzed at the 2-cell stage and the remaining embryos were cultured in KSOM medium and analyzed at subsequent preimplantation stages. In order to control the rate of parthenogenetic activation, the sperm suspension was heated to 56°C to disrupt the acrosome. Treated sperms were found to have no motility and disrupted plasma membrane upon dual staining with 4,6-diamidino-2-phenylindole (DAPI) and propidium iodide (PI). The heated sperm suspension was then used in the same manner as untreated sperms described above.

### Vital Stain

In order to observe viability of spermatozoa, they were collected and were mixed with an equal volume of the vital staining solution (eosin Y 0.1 g, fast green 0.2 g and DDW 100 ml). After 3 to 5 min of incubation at room temperature, viability of spermatozoa was observed.

### Sperm Motility

Sperm motility was determined by microscopic observation, which both percentages of motile sperm and the rate of forward movement were assessed on a scale 0 to 4 (0 absent, 1 weak, 2 definite, 3 good, and 4 vigorous).

### Immunolabeling and Flow Cytometry Analyses

Animals from a litter were analyzed in parallel when they were 5 weeks old. Thymus and spleen were isolated, washed in Dulbecco’s modified Eagle’s medium (DMEM) and was broken up using a homogenizer. Cells were counted and washed twice in PBS. 1×10^6^ cells were transferred to fluorescence-activated cell sorter tubes and labeled with an antibody (Thymus: anti-CD8-FITC and anti-CD4-PE. Spleen: CD3-APC and B220-FITC). Cells were washed twice with ice-cold PBS and analyzed using a FACSVantage Flow Cytometer and CELLQuest software (BD Sciences, San Jose, CA, USA).

### Statistics

Data are represented as mean values with standard deviation. Student’s *t*-test and *X* ^2^ analyses were used to test for significant differences. Results were considered significantly different with a *p*<0.05.
